# UK medical school over-assessment: a simple solution

**DOI:** 10.1007/s40037-013-0088-6

**Published:** 2013-10-08

**Authors:** Neel Sharma

**Affiliations:** Barts and the London School of Medicine and Dentistry, London, UK

Dear Editor

Assessment is rife in UK medical schools. Students are tested annually or biannually, as the case may be, in written and practical form. This allows for assessment of clinical detail and demonstration of hands-on competence, which is governed typically by the Medical Schools Council.

This year saw the launch of the situational judgement test, designed to assess final-year students on an array of FY1 professional attributes. It also saw the piloting of the prescribing skills assessment based on concerns of prescribing errors and lack of prescribing confidence. Both these assessments are unique to UK medical training.

I was fortunate not to have to undergo such assessment. But with media highlights of ‘Black Wednesday’ in the 1st week of August, it is clear our exiting students are not up to scratch. I would have branded myself such when I started working back in 2007.

So what is the solution and why are we still getting it wrong? After all, many an educationalist can draft a strong case for assessment and the potential benefits for doing so. But, from a personal perspective, we simply are not addressing the obvious—adequate patient exposure coupled with adequate practice. Yes, it may seem strange to suggest the obvious but most things in life are conquered through active pursuit. No one learns to drive by reading the car manual and no one would dare fly a plane by simple observation. We have put too much emphasis on self-directed book work and the cluster by the bedside approach without allowing our students to simply ‘do’. Now before heads turn and foreheads frown, I’m not suggesting our students should be able to perform the complexities of brain surgery. Rather a more active undertaking of what is required at foundation level. So, with appropriate supervision why don’t we let our students undertake procedural skills on actual patients rather than rely on a plastic models, and let them document a management plan or prescribe accordingly for example.

Unfortunately these aspects are eventually learned on the job with some trainees never being exposed to more complex procedures until their speciality training. We should all appreciate that educating the next generation does not exist at all, rather a hand-holding process which will leave them unskilled for many years to come.

